# Machine learning-based prediction of lymph node metastasis in papillary thyroid carcinoma: an interpretable multidimensional model

**DOI:** 10.3389/fendo.2026.1866805

**Published:** 2026-09-28

**Authors:** Xiaoqing Yuan, Yahui Wu, Yixiang Xing, Min Kang, Ke Lei, Qihang Gao, Yun Shen

**Affiliations:** 1 Department of Pathology, Tongling People’s Hospital, Tongling, Anhui, China; 2 Tongling Academy of Medical Sciences, Tongling, Anhui, China; 3 Department of Pathology, Heping Hospital Affiliated to Changzhi Medical College, Changzhi, Shanxi, China

**Keywords:** lymph node metastasis, machine learning, papillary thyroid carcinoma, predictive modeling, SHAP interpretability, support vector machine

## Abstract

**Background:**

Lymph node metastasis (LNM) is a prevalent feature in papillary thyroid carcinoma (PTC) that significantly influences surgical management and prognosis. Accurate identification of LNM risk is essential for optimizing adjuvant therapy and long-term surveillance strategies in clinical practice.

**Methods:**

This retrospective study analyzed 232 patients who underwent thyroidectomy. We employed LASSO regression to select six predictive features from a comprehensive covariate panel. Subsequently, seven machine learning algorithms were trained and evaluated via grid search and 5-fold cross-validation. The optimal model was interpreted using SHAP analysis to elucidate feature contributions.

**Results:**

LASSO-based feature selection retained six predictive features: *BRAF* mutation status, tumor size, capsular invasion, extrathyroidal extension, multifocality, and TSH. Among the seven algorithms, the Support Vector Machine (SVM) model showed the most favorable validation performance, achieving an AUC of 0.849 (95% CI, 0.756–0.934) and an accuracy of 0.768 in the validation cohort. SHAP analysis identified TSH and tumor size as the most influential contributors to model predictions, enabling transparent and individualized LNM risk stratification.

**Conclusion:**

The SVM-based model demonstrated favorable internal validation performance and provided an interpretable framework for individualized LNM risk stratification in patients with PTC. Further prospective multicenter validation is required before routine clinical implementation.

## Introduction

Papillary thyroid carcinoma (PTC) is the most common histological subtype of thyroid cancer and generally has a favorable long-term prognosis (1, 2). Nevertheless, cervical lymph node metastasis (LNM) is common in PTC and represents an important determinant of clinical management. The presence of LNM has been associated with an increased risk of locoregional recurrence and can influence postoperative risk stratification, surveillance intensity, and subsequent management strategies (2–4). Therefore, accurate assessment of cervical nodal status remains an important component of individualized management in patients with PTC.

Accurate identification of cervical LNM, however, remains challenging. Preoperative ultrasonography is the principal imaging modality for cervical lymph-node assessment, but its sensitivity for metastatic lymph nodes is imperfect and may vary according to nodal compartment, lesion characteristics, and operator-dependent interpretation. A systematic review and meta-analysis reported a pooled patient-based sensitivity of 0.41 and specificity of 0.92 for ultrasonography in detecting cervical LNM in PTC (5). Postoperative histopathological examination remains the definitive method for confirming LNM in surgically removed lymph nodes; however, the completeness of pathological nodal assessment may be influenced by the extent of lymph-node dissection and nodal sampling (2). These limitations highlight the need for complementary and objective risk-stratification approaches. Machine learning (ML) is well suited to this task because it can integrate multidimensional information and capture complex nonlinear relationships and interactions among predictors that may not be fully represented by conventional statistical approaches (6, 7).

Despite the increasing number of prediction models for LNM in PTC, their discriminatory performance and generalizability remain variable. A 2024 meta-analysis of 107 studies involving 136,245 patients reported pooled c-indices of 0.762 (95% CI, 0.747–0.777) in training datasets and 0.773 (95% CI, 0.754–0.791) in validation datasets (8). A subsequent systematic review and meta-analysis of 35 studies evaluating machine-learning and deep-learning approaches for thyroid cancer metastasis reported an overall AUC-ROC of 0.818, but also demonstrated substantial methodological and clinical heterogeneity across studies (9). Similar variability has been observed in individual prediction studies. Luo et al. reported an AUROC of 0.816 in the training cohort, which decreased to 0.749–0.777 across three external validation cohorts (10), while Hao et al. reported a test-set AUC of 0.798 for their machine-learning model (11). Furthermore, several representative prediction models have relied predominantly on clinical and ultrasonographic characteristics, and the incremental predictive contribution of molecular markers remains uncertain. Notably, a recent study found that the addition of *BRAF*^V600E^ mutation status did not significantly improve the diagnostic performance of an LNM prediction model (12). These findings underscore the need for multidimensional and interpretable prediction frameworks while also emphasizing that the added value of individual molecular markers should be evaluated rather than assumed.

To address these gaps, the present study employed LASSO-based feature selection and systematically compared seven predictive algorithms for pathologically detected cervical LNM in patients with PTC. The final model was selected according to its internal validation performance and was further interpreted using SHAP analysis to characterize the contribution of individual features to model predictions. In addition, an ablation analysis was performed to specifically evaluate the incremental predictive contribution of *BRAF*^V600E^ mutation status. The aim of this study was to develop an interpretable multidimensional model for individualized postoperative LNM risk stratification while explicitly evaluating the added contribution of molecular information.

## Materials and methods

### Study design and population

This retrospective cohort study was conducted at Tongling People’s Hospital, encompassing patients who underwent thyroid surgery between January 2023 and April 2026. The diagnosis of papillary thyroid carcinoma (PTC) was histologically confirmed by two independent senior pathologists, adhering to the World Health Organization (WHO) classification system, which remains the diagnostic gold standard (13, 14). To ensure data integrity, we implemented stringent exclusion criteria: (1) patients aged <18 years; (2) non-PTC histological subtypes; (3) absence of *BRAF* mutation testing; and (4) incomplete clinical, laboratory, or pathological data required for model development and outcome ascertainment. After exclusion of non-malignant tumors and cases with unclear pathological diagnoses, 729 patients remained eligible for further screening. Of these, 468 patients were excluded because *BRAF*^V600E^ testing was unavailable, leaving 261 patients with documented *BRAF* results. A further 29 patients were excluded because of incomplete clinical or biochemical data required for model development, resulting in a final analytic cohort of 232 patients (**Figure 1**).

**Figure 1:**
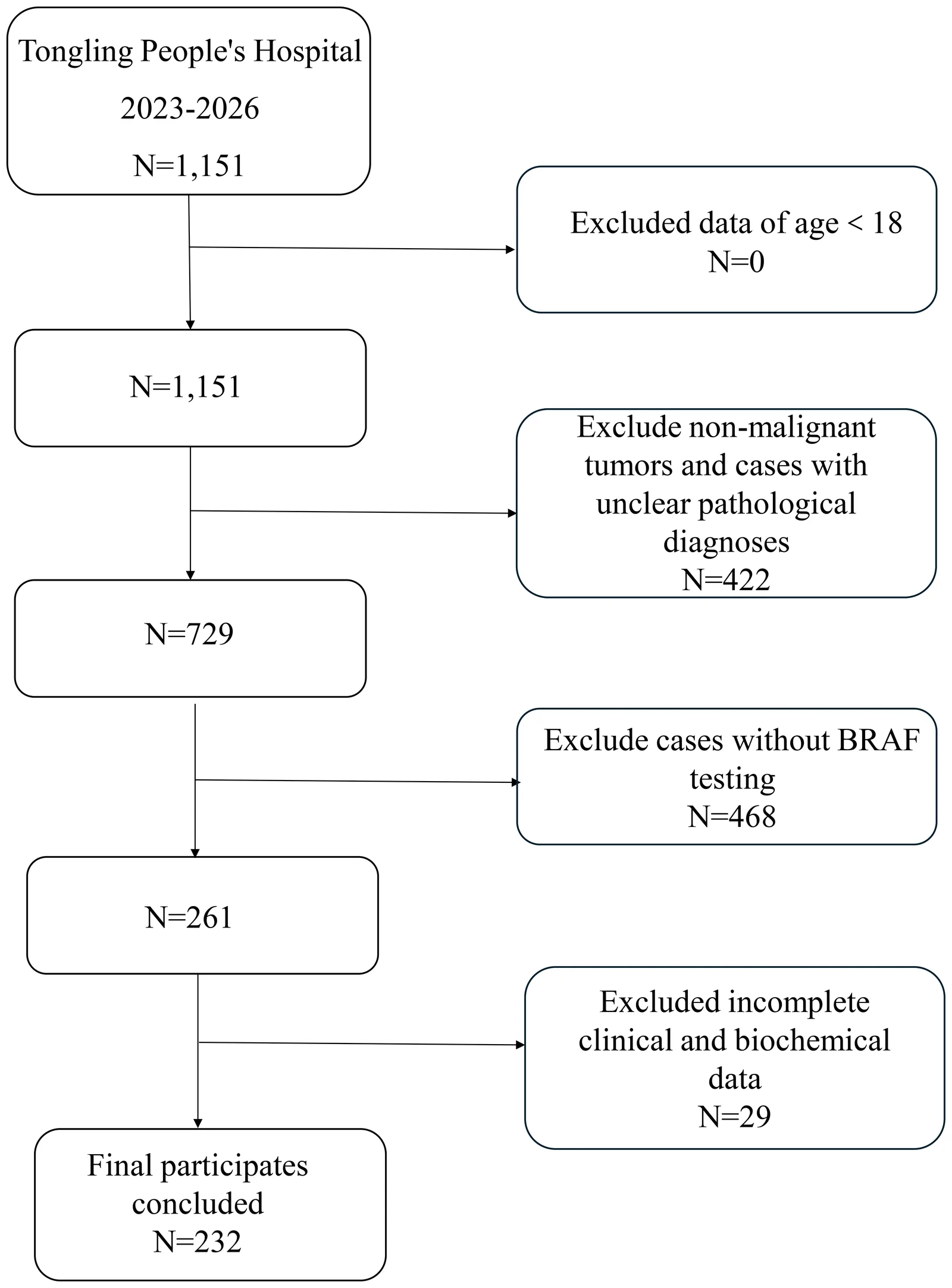
Flowchart of patient inclusion and exclusion. A total of 1,151 patients undergoing thyroid surgery were initially screened. After excluding 422 patients with non-malignant tumors or unclear pathological diagnoses, 729 patients remained. Among these, 468 patients were excluded because *BRAF*^V600E^ testing was unavailable, leaving 261 patients with available *BRAF* results. A further 29 patients were excluded because of incomplete clinical or biochemical data, resulting in a final analytic cohort of 232 patients. No patients were excluded because of age <18 years.

Surgical procedures included total thyroidectomy and lobectomy/hemithyroidectomy according to individual clinical indications. Among the 232 included patients, 105 (45.26%) underwent total thyroidectomy and 127 (54.74%) underwent lobectomy/hemithyroidectomy. Regarding cervical lymph-node surgery, 189 patients (81.47%) underwent central compartment lymph-node dissection only, whereas 43 patients (18.53%) underwent combined central and lateral compartment lymph-node dissection. The extent of thyroidectomy and lymph-node dissection was determined by the treating surgical team according to preoperative imaging, intraoperative findings, tumor characteristics, and routine institutional practice.

### Outcome and predictors

The primary outcome of this study was pathologically detected cervical lymph node metastasis (LNM), defined according to postoperative histopathological examination of the lymph nodes removed during surgery and recorded as either present or absent. Because the anatomical extent of lymph-node dissection was determined by the surgical procedure performed, the endpoint reflects LNM detected in the resected nodal specimens rather than a standardized comprehensive assessment of all cervical lymph-node compartments. In accordance with established oncological guidelines, published risk-factor studies, and routine clinical availability, a total of 32 candidate predictors were prespecified to provide a multidimensional assessment (2, 15–18). Tumor size was categorized as ≤1 cm, >1–2 cm, >2–4 cm, and >4 cm, and age was dichotomized at 55 years according to the AJCC 8th edition staging framework (19). Clinicopathological and molecular variables additionally included *BRAF*^V600E^ mutation status, multifocality, capsular invasion, neural invasion, vascular invasion, and extrathyroidal invasion. General clinical and metabolic covariates included sex, BMI, smoking and alcohol history, hypertension, and diabetes. These variables were considered candidate covariates rather than established independent risk factors and were subsequently subjected to LASSO-based feature selection. Laboratory variables included Tg, TSH, TgAb, CEA, systemic inflammatory indices (NLR, PLR, and LMR), WBC, neutrophil and lymphocyte counts, platelet count, albumin, LDH, fasting glucose, TC, TG, LDL, and HDL. Preoperative ultrasonography is an essential component of routine cervical lymph-node assessment; however, standardized nodal ultrasound descriptors were not available as structured variables in the analytic dataset used for the present model and therefore were not entered into feature selection (5). Hashimoto’s thyroiditis was likewise not included as a standardized structured candidate variable; published studies have reported heterogeneous associations between Hashimoto’s thyroiditis and LNM in PTC (15, 20). Accordingly, the present model should be regarded as complementary to conventional imaging rather than as a replacement for preoperative ultrasonography.

### Data preprocessing

Data cleaning and preparation were performed to ensure robust model performance. The distribution of missing data and the subsequent imputation workflow are illustrated in **Figure 2**. We addressed these gaps using multiple imputation to preserve sample size and mitigate potential bias associated with case-wise deletion (21). For reproducibility, we generated five imputed datasets using a fixed random seed. Before feature selection and model development, categorical variables were converted to numerical codes using predefined coding rules. Binary variables, including *BRAF* mutation status, capsular invasion, neural invasion, vascular invasion, extrathyroidal invasion, multifocality, smoking history, alcohol history, diabetes, and hypertension, were represented as binary indicators (0 = absent/no and 1 = present/yes). Tumor size was treated as an ordered categorical variable and encoded as 1 (≤1 cm), 2 (>1–2 cm), 3 (>2–4 cm), and 4 (>4 cm), preserving its inherent ordinal structure; therefore, one-hot encoding was not applied to this variable. The same coding scheme was consistently applied throughout feature selection and model development. Continuous variables retained their original measurement scales, whereas binary and ordinal categorical variables retained their predefined numerical coding throughout model development (22).

**Figure 2:**
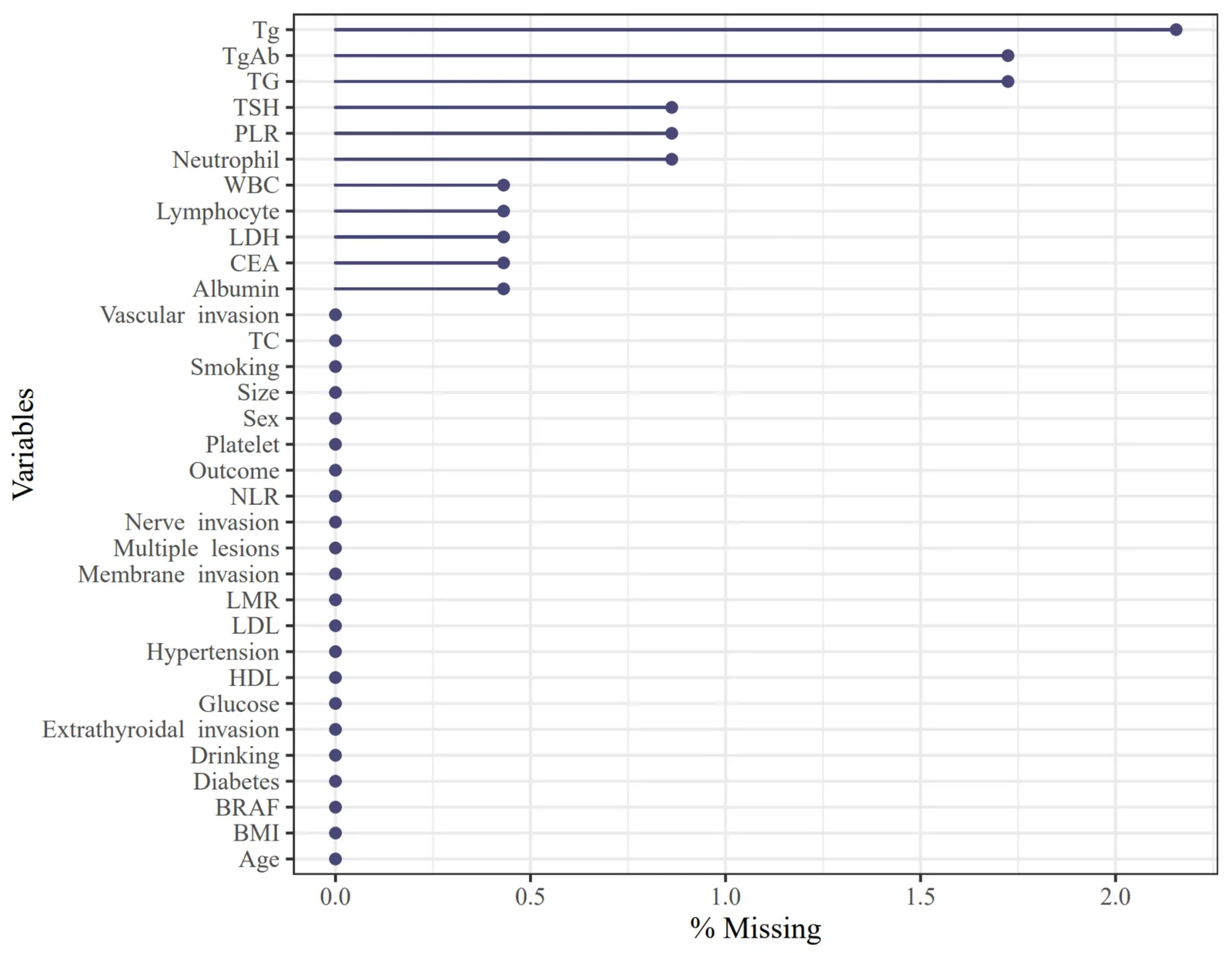
Missing data distribution in the dataset. Percentage of missing values for each variable. The chart displays the frequency of missing data across the collected clinical, biochemical, and pathological indicators.

The dataset was then partitioned into a training cohort (70%) and a validation cohort (30%), with stratification according to lymph node metastasis status to preserve the outcome distribution across the two subsets. To optimize feature selection, we employed Least Absolute Shrinkage and Selection Operator (LASSO) regression with a binomial family and L1 regularization (23). LASSO feature selection was performed exclusively in the training cohort, and the validation cohort was held out and was not involved in feature selection or subsequent hyperparameter tuning. This approach utilized 10-fold cross-validation across 1,000 candidate lambda values to select a parsimonious subset of predictive features for lymph node metastasis while reducing high-dimensional noise. The LASSO-retained feature set was subsequently used as input for seven machine-learning architectures, including Logistic Regression, Decision Tree, Support Vector Machine, Artificial Neural Network, LightGBM, XGBoost, and Random Forest.

Finally, the predictive efficacy and clinical utility of these models were rigorously assessed through a comprehensive analytical framework, incorporating receiver operating characteristic (ROC) curves, calibration plots, and decision curve analysis (DCA) to validate both discriminatory power and real-world applicability.

### Machine learning algorithms

To develop the predictive framework for lymph node metastasis, we evaluated seven algorithms: Logistic Regression (LR), Decision Tree (DT), Random Forest (RF), Support Vector Machine (SVM), Artificial Neural Network (ANN), XGBoost, and LightGBM. LR was used as the baseline statistical model and was not subjected to hyperparameter tuning. For the remaining algorithms, hyperparameter optimization was performed in the training cohort using exhaustive grid search combined with 5-fold cross-validation, with the mean cross-validated AUC used as the optimization criterion. The validation cohort was not used for either LASSO feature selection or hyperparameter optimization. The complete candidate search spaces and the final selected hyperparameters are provided in **Supplementary Table 1**. The optimal configurations were as follows: DT (ccp_alpha = 0.01, max_depth = None, max_features = ‘sqrt’, min_samples_split = 5); RF (n_estimators = 50, max_features = 2); XGBoost (learning_rate = 0.2, max_depth = 3, n_estimators = 50, subsample = 0.6); LightGBM (colsample_bytree = 1.0, learning_rate = 0.1, n_estimators = 100, num_leaves = 31, subsample = 0.6); SVM (C = 0.1, degree = 2, gamma = 0.01, kernel = ‘rbf’); and ANN (activation = ‘tanh’, hidden_layer_sizes = (10, 10), alpha = 0.001, batch_size = 16, learning_rate_init = 0.005).

An additional ablation analysis was performed to quantify the incremental predictive contribution of *BRAF*^V600E^ mutation status. Using the same training and validation cohorts, the final six-variable SVM model was compared with a reduced five-variable SVM in which *BRAF*^V600E^ status was removed while the remaining selected predictors were retained. After removal of *BRAF*, the reduced SVM was re-tuned exclusively within the training cohort using the same hyperparameter search space and 5-fold cross-validation procedure as the full model. AUCs were evaluated in the held-out validation cohort, with 95% CIs estimated using 1,000 bootstrap resamples. The paired difference between the AUCs of the full and reduced models was assessed using DeLong’s test.

### Model evaluation

The predictive performance and clinical utility of the seven machine learning architectures were evaluated through a multifaceted analytical framework. Discrimination was quantified by the area under the ROC curve (AUC), and corresponding 95% confidence intervals (CIs) were estimated using 1,000 bootstrap resamples in both the training and validation cohorts. To assess the reliability of predicted probabilities, calibration curves were constructed by comparing observed outcome frequencies with predicted risk probabilities; calibration performance was further quantified using the Brier score, with lower values indicating better agreement.

Given that traditional performance metrics often fail to capture clinical utility, DCA was employed to calculate the net benefit of each model across a range of threshold probabilities. This approach allowed for a comprehensive assessment of the clinical value of the models by balancing the trade-offs between true-positive detections and false-positive interventions.

### SHAP interpretability analysis

To enhance the transparency of the optimal Support Vector Machine model, we conducted a comprehensive SHAP (SHapley Additive exPlanations) analysis based on game theory. Global interpretability was initially established using a bar plot to rank the absolute mean SHAP values, identifying the relative contribution of each feature to the overall model prediction. This was further refined through a beeswarm plot, which illustrates the distribution and impact direction (positive or negative) of these features on LNM risk. Beyond global patterns, we performed instance-level analysis using waterfall plots and force plots for individual cases, which elucidate the specific contribution of each feature toward the predicted risk probability for a single patient. This dual-layered interpretability framework effectively bridges the gap between the “black-box” nature of machine learning and clinical decision-making, providing a transparent basis for personalized risk assessment.

### Statistical analysis

Statistical computation and data orchestration were conducted using R version 4.5.1 for preprocessing, while model training and interpretability analyses were executed in Python version 3.10. Missing data were handled using multiple imputation by chained equations (MICE), generating five imputed datasets under the missing at random assumption. Continuous descriptors are expressed as mean ± standard deviation for normally distributed variables with equal variances, or as median (interquartile range) for non-normally distributed data; corresponding group disparities were evaluated using the Student’s t-test or the Wilcoxon rank-sum test. Categorical variables are summarized as frequencies and proportions, with group differences assessed via Pearson’s χ2 test or Fisher’s exact test where appropriate. All tests were two-tailed, and statistical significance was strictly defined as a P-value below 0.05.

## Results

### Patient characteristics

The baseline clinical and pathological characteristics of the 232 enrolled patients are summarized in **Table 1**. Among these participants, 145 patients (62.50%) were diagnosed with lymph node metastasis (LNM), while 87 patients (37.50%) were classified into the non-LNM group. Baseline analysis revealed statistically significant differences between the two groups across several clinico-pathological and biochemical markers. Specifically, LNM was significantly associated with positive *BRAF* mutation status (86.21% vs 67.82%, P = 0.001), larger tumor size (P < 0.001), presence of capsular invasion (51.03% vs 24.14%, P < 0.001), extrathyroidal invasion (8.28% vs 1.15%, P = 0.04), and multifocality (33.79% vs 17.24%, P = 0.01). Regarding biochemical profiles, patients with LNM demonstrated significantly elevated levels of TSH (2.44 vs 1.76 mIU/L, P < 0.001), fasting glucose (5.79 vs 5.47 mmol/L, P = 0.015), and LDH (196.23 vs 183.01 U/L, P = 0.006). Furthermore, systemic inflammatory indices, including NLR (2.55 vs 2.06, P < 0.001) and PLR (144.24 vs 115.63, P < 0.001), were notably higher in the LNM group, whereas LMR was significantly lower (3.85 vs 4.51, all P < 0.001). No significant differences were observed regarding age, sex, BMI, metabolic lipid profiles, extent of thyroidectomy, or extent of lymph-node dissection (all P > 0.05).

**Table 1:** Baseline characteristics of the participants.

Variables	Overall (N = 232)	No lymph node metastasis (N = 87)	Lymph node metastasis (N = 145)	*P*
Sex, Male [n (%)]	55 (23.71)	24 (27.59)	31 (21.38)	0.36
Age, ≥55 [n (%)]	94 (40.52)	34 (39.08)	60 (41.38)	0.84
*BRAF* mutation status, yes [n (%)]	184 (79.31)	59 (67.82)	125 (86.21)	**0.001**
Tumor size, [n (%)]				**<0.001**
≤1 cm	145 (62.50)	73 (83.91)	72 (49.66)	
>1–2 cm	68 (29.31)	13 (14.94)	55 (37.93)	
>2–4 cm	12 (5.17)	0 (0.00)	12 (8.28)	
>4 cm	7 (3.02)	1 (1.15)	6 (4.14)	
Capsular invasion, yes [n (%)]	95 (40.95)	21 (24.14)	74 (51.03)	**<0.001**
Nerve invasion, yes [n (%)]	3 (1.29)	0 (0.00)	3 (2.07)	0.54
Vascular invasion, yes [n (%)]	4 (1.72)	0 (0.00)	4 (2.76)	0.30
Extrathyroidal invasion, yes [n (%)]	13 (5.60)	1 (1.15)	12 (8.28)	**0.04**
Multifocality, yes [n (%)]	64 (27.59)	15 (17.24)	49 (33.79)	**0.01**
BMI, [n (%)]				0.79
Normal weight	108 (46.55)	43 (49.43)	65 (44.83)	
Overweight	81 (34.91)	29 (33.33)	52 (35.86)	
Obese	43 (18.53)	15 (17.24)	28 (19.31)	
Smoking, yes [n (%)]	73 (31.47)	23 (26.44)	50 (34.48)	0.26
Drinking, yes [n (%)]	59 (25.43)	17 (19.54)	42 (28.97)	0.15
Diabetes history, yes [n (%)]	38 (16.38)	12 (13.79)	26 (17.93)	0.52
Hypertension history, yes [n (%)]	47 (20.26)	18 (20.69)	29 (20.00)	0.98
Extent of thyroidectomy, n (%)				0.81
Lobectomy/hemithyroidectomy	127 (54.74)	49 (56.32)	78 (53.79)	
Total thyroidectomy	105 (45.26)	38 (43.68)	67 (46.21)	
Extent of lymph-node dissection, n (%)				0.90
Central compartment only	189 (81.47)	70 (80.46)	119 (82.07)	
Central & lateral compartment	43 (18.53)	17 (19.54)	26 (17.93)	
TSH (mIU/L)^†^	2.19 (0.66)	1.76 (0.48)	2.44 (0.63)	**<0.001**
Tg (ng/mL)^‡^	27.93 [19.22, 39.70]	19.68 [13.38, 25.37]	35.24 [26.84, 45.35]	**<0.001**
TgAb (IU/mL)^‡^	37.72 [26.46, 60.06]	37.97 [27.32, 59.16]	37.28 [25.04, 60.38]	0.71
CEA (ng/mL)^†^	2.74 (0.96)	2.59 (1.00)	2.84 (0.92)	0.06
NLR^†^	2.36 (0.72)	2.06 (0.71)	2.55 (0.66)	**<0.001**
PLR^†^	133.51 (27.90)	115.63 (22.77)	144.24 (25.10)	**<0.001**
LMR^†^	4.10 (0.92)	4.51 (0.89)	3.85 (0.86)	**<0.001**
WBC (×10^9^/L)^†^	6.82 (1.24)	6.63 (1.38)	6.93 (1.13)	0.07
Neutrophil (×10^9^/L)^†^	4.43 (0.85)	4.33 (0.98)	4.48 (0.76)	0.20
Lymphocyte (×10^9^/L)^†^	1.85 (0.44)	1.82 (0.47)	1.87 (0.43)	0.36
Platelet (×10^9^/L)^†^	249.14 (53.55)	239.43 (55.29)	254.96 (51.80)	**0.032**
Albumin (g/L)^†^	41.00 (2.87)	41.32 (2.58)	40.81 (3.03)	0.19
LDH (U/L)^†^	191.27 (35.93)	183.01 (34.00)	196.23 (36.25)	**0.006**
Glucose (mmol/L)^†^	5.67 (1.00)	5.47 (1.02)	5.79 (0.97)	**0.015**
TC (mmol/L)^†^	4.90 (0.92)	4.84 (0.92)	4.93 (0.92)	0.47
TG (mmol/L)^‡^	1.54 [1.06, 2.02]	1.45 [1.02, 1.89]	1.58 [1.11, 2.10]	0.18
LDL (mmol/L)^†^	2.86 (0.71)	2.94 (0.73)	2.81 (0.69)	0.17
HDL (mmol/L)^†^	1.28 (0.29)	1.33 (0.30)	1.25 (0.28)	0.07

Categorical variables are presented as n (%). For dichotomous presence/absence variables, only one prespecified category is displayed: male for sex, ≥55 years for age, and the “Yes” or presence category for the remaining binary clinical variables. Procedural variables, including extent of thyroidectomy and extent of lymph-node dissection, are presented with all mutually exclusive categories. Percentages were calculated using the corresponding column totals.

† Data are presented as mean (standard deviation [SD]).

‡ Data are presented as median [interquartile range (IQR)]. Between-group comparisons were performed using Student’s t-test for normally distributed continuous variables, the Wilcoxon rank-sum test for non-normally distributed continuous variables, and Pearson’s χ^2^ test or Fisher’s exact test for categorical variables, as appropriate. All P values were two-sided, and P < 0.05 was considered statistically significant.

BMI, body mass index; *BRAF*, B-Raf proto-oncogene; CEA, carcinoembryonic antigen; HDL, high-density lipoprotein cholesterol; IQR, interquartile range; LDH, lactate dehydrogenase; LDL, low-density lipoprotein cholesterol; LMR, lymphocyte-to-monocyte ratio; LNM, lymph node metastasis; NLR, neutrophil-to-lymphocyte ratio; PLR, platelet-to-lymphocyte ratio; SD, standard deviation; TC, total cholesterol; Tg, thyroglobulin; TgAb, thyroglobulin antibody; TG, triglyceride; TSH, thyroid-stimulating hormone; WBC, white blood cell. Bold values indicate statistical significance (P < 0.05).

To evaluate potential selection bias associated with the exclusion of patients without *BRAF*^V600E^ testing, we performed an additional comparison between the final analytic cohort and patients excluded specifically because *BRAF* testing was unavailable. Among the 729 patients remaining after exclusion of non-malignant tumors and cases with unclear pathological diagnoses, 468 lacked *BRAF* testing, leaving 261 patients with available *BRAF* results; 29 of these patients were subsequently excluded because of incomplete clinical or biochemical data, resulting in the final analytic cohort of 232 patients. Pathological LNM was observed in 145 of 232 patients (62.5%) in the final analytic cohort and in 282 of 468 patients (60.3%) excluded because *BRAF* testing was unavailable. The difference in LNM prevalence was not statistically significant (P = 0.567; **Supplementary Table 2**).

### Predictor selection

To identify a parsimonious subset of predictive features, we applied LASSO regression to the candidate covariates in the training cohort. Six variables were retained at the λ value corresponding to the one-standard-error criterion (λ1se): *BRAF* mutation status, tumor size, capsular invasion, extrathyroidal invasion, multifocality, and TSH (**Figure 3A**). In **Figure 3B**, the vertical dotted lines indicate λmin and λ1se rather than predictor-specific cutoff values. These LASSO-retained variables were subsequently used as input features for the seven machine-learning algorithms. Because LASSO is a penalized feature-selection method, these variables are described as selected predictive features rather than independent prognostic or causal factors.

**Figure 3:**
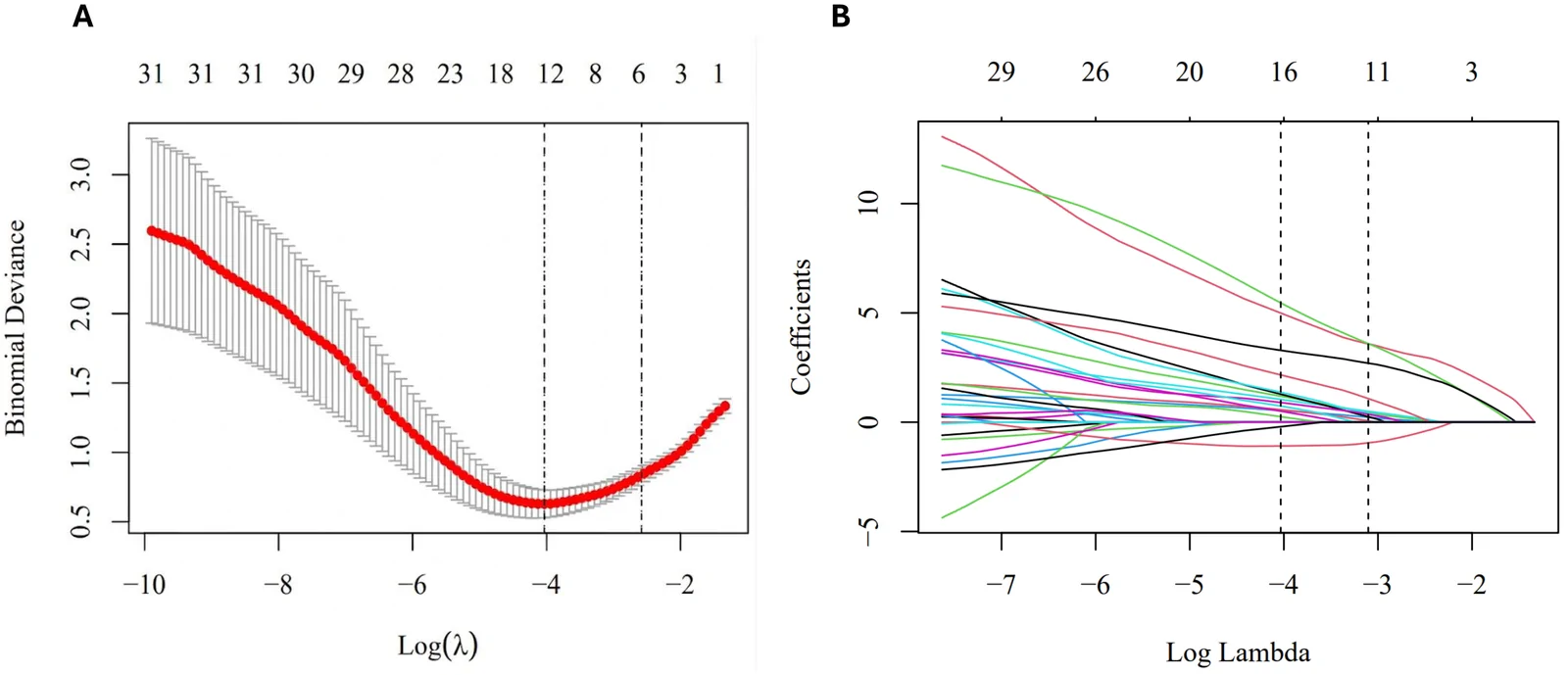
LASSO feature selection. **(A)** Ten-fold cross-validation curve showing binomial deviance across log(lambda), with lambda_min and lambda_1se indicated by vertical dotted lines. **(B)** Coefficient paths of candidate variables across log(lambda). Six predictive features - BRAF mutation status, tumor size, capsular invasion, extrathyroidal invasion, multifocality, and TSH - were retained at the lambda_1se threshold.

### Multimodel integrated analysis for classification

The performance of seven machine learning algorithms was systematically evaluated in the training and validation cohorts (**Tables 2**, **3**). Although Random Forest and XGBoost showed strong discrimination in the training cohort, their performance decreased in the validation cohort, suggesting potential overfitting. In contrast, the SVM model achieved the highest validation AUC among the evaluated models and showed a favorable balance across multiple performance metrics. Specifically, the SVM model achieved an AUC of 0.912 (95% CI, 0.864–0.950) in the training cohort and 0.849 (95% CI, 0.756–0.934) in the validation cohort, with corresponding accuracies of 0.834 and 0.768, respectively.

**Table 2:** Performance metrics of machine learning models on the training set.

Model	AUC (95% CI)	Accuracy	Precision (PPV)	Sensitivity (Recall)	Specificity	F1 score	Kappa	Youden’s J	NPV
Logistic	0.926 (0.885–0.961)	0.85	0.88	0.87	0.80	0.88	0.67	0.68	0.79
Decision Tree	0.911 (0.863–0.952)	0.88	0.90	0.90	0.84	0.90	0.74	0.74	0.84
Random Forest	0.998 (0.995–1.000)	0.99	1.00	0.98	1.00	0.99	0.97	0.98	0.97
XGBoost	0.976 (0.953–0.993)	0.93	0.94	0.94	0.90	0.94	0.84	0.84	0.90
LightGBM	0.976 (0.953–0.992)	0.94	0.95	0.95	0.92	0.95	0.87	0.87	0.92
SVM	0.912 (0.864–0.950)	0.83	0.92	0.80	0.89	0.86	0.66	0.69	0.73
ANN	0.899 (0.850–0.943)	0.84	0.85	0.91	0.72	0.88	0.65	0.63	0.83

AUC values are reported with corresponding 95% confidence intervals (CIs). AUC, area under the receiver operating characteristic curve; PPV, positive predictive value; NPV, negative predictive value. Precision and PPV are equivalent and were calculated as TP/(TP + FP). Sensitivity and Recall are equivalent and were calculated as TP/(TP + FN). Specificity was calculated as TN/(TN + FP), and NPV as TN/(TN + FN). Accuracy was calculated as (TP + TN)/(TP + TN + FP + FN). The F1 score represents the harmonic mean of Precision and Recall. Cohen’s kappa quantifies classification agreement beyond chance, and Youden’s J was calculated as Sensitivity + Specificity − 1. TP, true positive; TN, true negative; FP, false positive; FN, false negative.

**Table 3:** Performance metrics of machine learning models on the validation set.

Model	AUC (95% CI)	Accuracy	Precision (PPV)	Sensitivity (Recall)	Specificity	F1 score	Kappa	Youden’s J	NPV
Logistic	0.835 (0.732–0.927)	0.74	0.74	0.91	0.46	0.81	0.40	0.37	0.75
Decision Tree	0.524 (0.380–0.677)	0.61	0.67	0.72	0.42	0.70	0.15	0.14	0.48
Random Forest	0.697 (0.563–0.817)	0.68	0.71	0.81	0.46	0.76	0.29	0.28	0.60
XGBoost	0.788 (0.685–0.882)	0.67	0.67	0.91	0.27	0.77	0.20	0.18	0.64
LightGBM	0.778 (0.669–0.878)	0.68	0.71	0.84	0.42	0.77	0.28	0.26	0.61
SVM	0.849 (0.756–0.934)	0.77	0.79	0.86	0.62	0.82	0.49	0.48	0.73
ANN	0.823 (0.716–0.915)	0.72	0.72	0.91	0.42	0.80	0.36	0.33	0.73

AUC values are reported with corresponding 95% confidence intervals (CIs). AUC, area under the receiver operating characteristic curve; PPV, positive predictive value; NPV, negative predictive value. Precision and PPV are equivalent and were calculated as TP/(TP + FP). Sensitivity and Recall are equivalent and were calculated as TP/(TP + FN). Specificity was calculated as TN/(TN + FP), and NPV as TN/(TN + FN). Accuracy was calculated as (TP + TN)/(TP + TN + FP + FN). The F1 score represents the harmonic mean of Precision and Recall. Cohen’s kappa quantifies classification agreement beyond chance, and Youden’s J was calculated as Sensitivity + Specificity − 1. TP, true positive; TN, true negative; FP, false positive; FN, false negative.

The comprehensive evaluation is visualized in **Figure 4**, which presents the calibration curves, DCA, and ROC curves. As shown in the calibration plots, the SVM model demonstrated optimal alignment between predicted probabilities and observed outcomes in the validation set, yielding a superior Brier score. Furthermore, DCA revealed that the SVM model provided the highest net benefit across a wide range of threshold probabilities. Taken together, these findings supported the selection of SVM as the final model on the basis of its overall validation performance, calibration, and estimated clinical net benefit.

**Figure 4:**
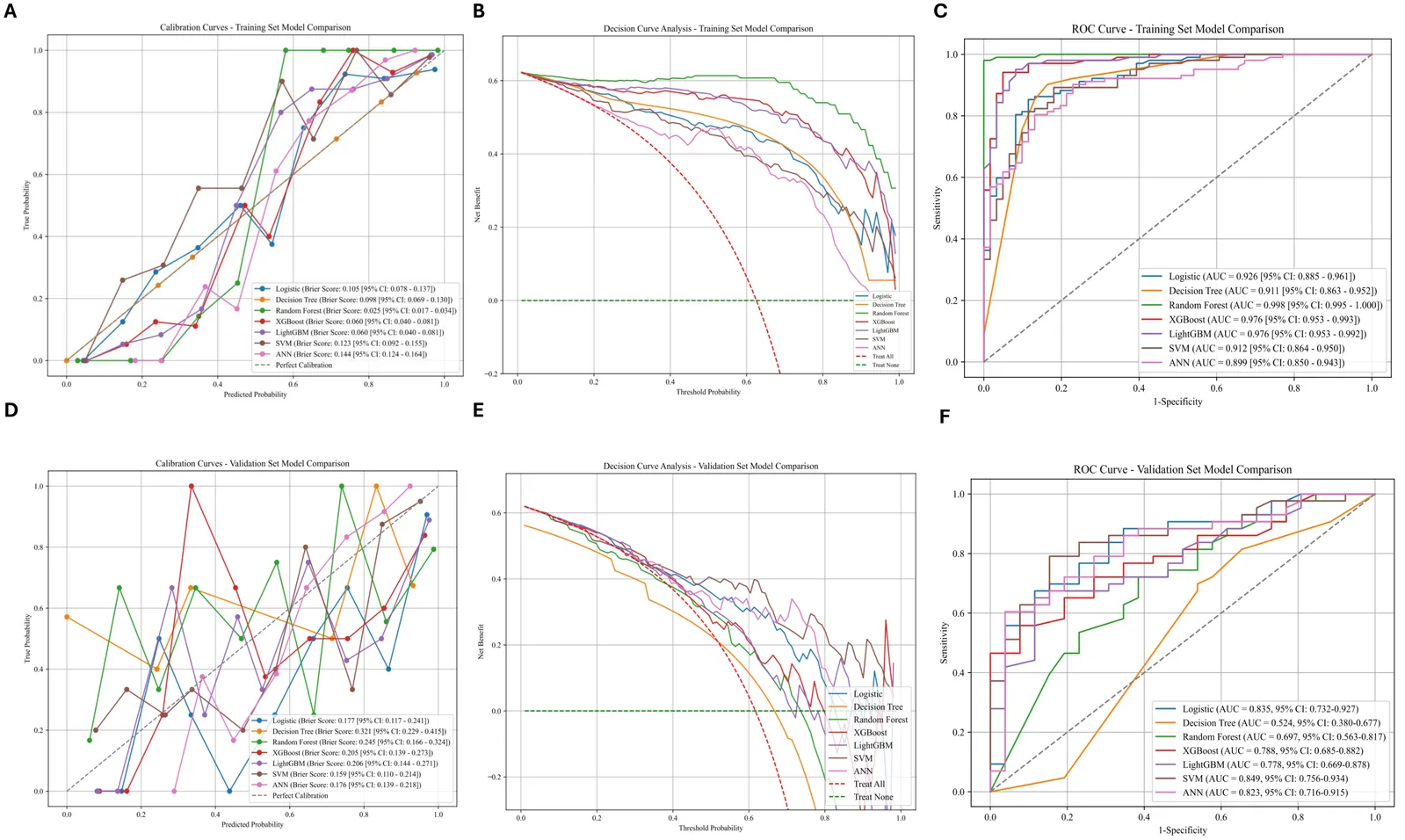
The performance and comparison of seven different predictive models. **(A)** Calibration curve for the training set. **(B)** DCA for the training set. **(C)** ROC curves for the training set. **(D)** Calibration curve for the validation set. **(E)** DCA for the validation set. **(F)** ROC curves for the validation set.

### Incremental contribution of *BRAF*^V600E^ mutation status

To evaluate the incremental contribution of *BRAF*^V600E^ mutation status, we performed an ablation analysis comparing the full six-variable SVM model with a reduced five-variable SVM excluding *BRAF*. The full model achieved a validation AUC of 0.849 (95% CI, 0.756–0.934), whereas the reduced model achieved an AUC of 0.843 (95% CI, 0.747–0.928). The absolute AUC difference was 0.007 and was not statistically significant by paired DeLong testing (P = 0.671). At a probability threshold of 0.50, the full and reduced models showed accuracies of 0.768 and 0.797, sensitivities of 0.860 and 0.860, and specificities of 0.615 and 0.692, respectively (**Supplementary Table 3**). These findings indicate that, although *BRAF*^V600E^ was retained during feature selection, its incremental contribution to validation discrimination was modest in this cohort.

### Interpretability and application of the model

To demystify the SVM model’s predictive mechanism, we utilized SHAP analysis to provide both global and local interpretability (**Figure 5**). **Figure 5A** displays the SHAP summary plot, where the distribution of red and blue points illustrates how high or low values of each predictor impact LNM risk. **Figure 5B** presents the feature importance ranking, confirming that TSH and tumor size are the most influential variables in driving model output. **Figure 5C** employs waterfall plots to elucidate the cumulative contribution of specific clinical features, detailing how each variable shifts an individual’s predicted probability from the baseline. Finally, **Figure 5D** provides force plots for specific clinical cases, visualizing the dynamic interplay between risk- enhancing factors (e.g., *BRAF* mutation) and protective attributes. This multi-layered interpretability framework effectively bridges the gap between complex machine learning architecture and clinical decision-making, offering a transparent and personalized approach to LNM risk stratification for PTC patients.

**Figure 5:**
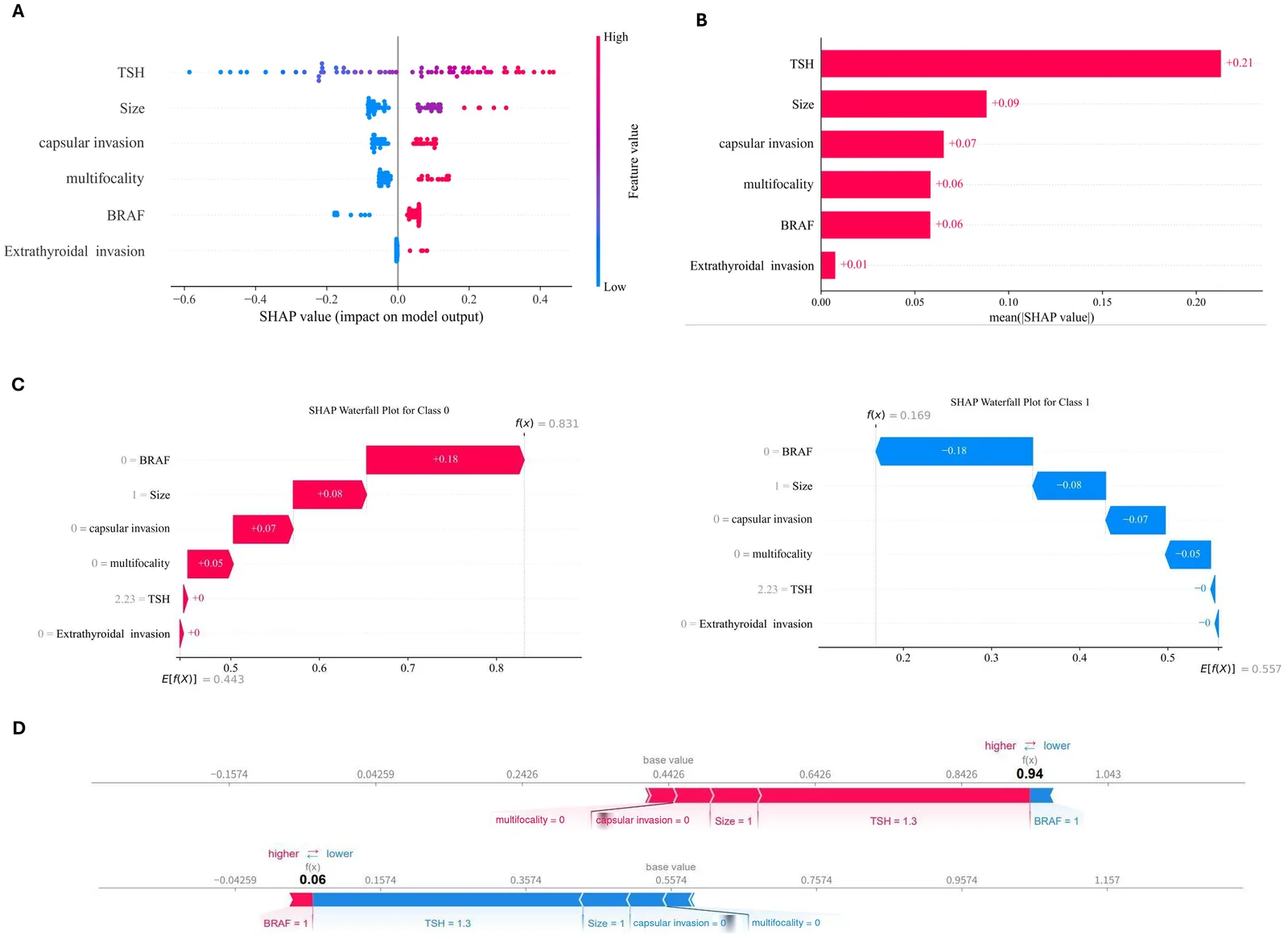
SHAP interpretability analysis of the optimal SVM model. **(A)** SHAP summary beeswarm plot showing global feature contributions, with red and blue dots representing higher and lower feature values, respectively. **(B)** Feature importance ranking based on mean absolute SHAP values. **(C)** Paired class-specific waterfall plots illustrating the cumulative contributions of variables for a single case, shown for class 0 and class 1. **(D)** Force plots depicting individual-level predictions for two cases, with red and blue indicating features that increase and decrease the model output, respectively. In the plots, Size denotes tumor size, Membrane invasion denotes capsular invasion, and Multiple lesions denotes multifocality.

## Discussion

This study developed and internally validated an interpretable machine learning-based risk stratification model for cervical lymph node metastasis (LNM) in patients with papillary thyroid carcinoma (PTC).

Previous studies have extensively explored factors associated with LNM in PTC, emphasizing the multifactorial nature of thyroid tumor progression. In the present study, LASSO-based feature selection retained six predictive features: *BRAF* mutation status, tumor size, capsular invasion, extrathyroidal extension (ETE), multifocality, and TSH. The biological and clinical plausibility of these features is supported by previous literature. For example, *BRAF*^V600E^ mutation has been associated with aggressive clinicopathological characteristics of PTC (24, 25), while tumor size, ETE, capsular invasion, multifocality, and TSH have also been linked to tumor aggressiveness or nodal involvement in previous studies (26, 27). Importantly, their retention by LASSO in the present analysis should be interpreted as evidence of predictive contribution within the model rather than as proof of independent causal or prognostic effects.

The incorporation of *BRAF*^V600E^ mutation status adds a molecular dimension to the present multidimensional prediction framework. Previous meta-analytic evidence has demonstrated an association between *BRAF*^V600E^ positivity and aggressive clinicopathological features of PTC, including LNM (28). However, an association with the outcome does not necessarily imply substantial incremental predictive value when the biomarker is added to a multivariable model. In our within-cohort ablation analysis, removal of *BRAF*^V600E^ resulted in only a modest decrease in validation AUC, from 0.849 to 0.843, and the paired difference was not statistically significant (P = 0.671; **Supplementary Table 3**). Therefore, although *BRAF*^V600E^ was retained during LASSO feature selection and may provide biologically relevant molecular information, the present data do not support attributing a major improvement in model performance to *BRAF* alone. This observation is also consistent with previous evidence showing that the incremental predictive value of *BRAF* may vary across clinical settings (12). Moreover, prediction models without *BRAF* in their final predictor sets have achieved moderate discrimination in independent cohorts (10, 11). Accordingly, the performance of our SVM model should be interpreted as arising from the integrated contribution of molecular, clinicopathological, and biochemical features rather than from any single predictor.

From a practical perspective, the proposed SVM model is intended as an adjunct to postoperative pathological assessment rather than as a replacement for histopathological diagnosis or guideline-based management. Once the six selected clinicopathological, biochemical, and molecular features are available, they can be entered into the model to generate an individualized estimate of LNM risk. The model may be particularly useful when pathological nodal assessment is limited or when the observed nodal findings are discordant with other clinicopathological features. In such situations, a high model-estimated risk may serve as an additional clinical signal prompting closer review of pathological specimens, targeted postoperative cervical imaging, or multidisciplinary reassessment. Conversely, a low model-estimated risk should not be used to exclude occult nodal disease. Therefore, the model should be viewed as a complementary risk-stratification tool that may help prioritize postoperative surveillance and further clinical review rather than as a stand-alone basis for determining radioactive iodine therapy, TSH suppression, reoperation, or other treatment decisions. Prospective multicenter studies are required to determine whether integration of the model into clinical workflows improves decision-making or patient outcomes.

An important finding of the present study was the substantial difference between training and validation performance among the evaluated algorithms. Random Forest achieved an AUC of 0.998 in the training cohort but decreased to 0.697 in the validation cohort, while XGBoost decreased from 0.976 to 0.788. These marked training–validation gaps indicate that the highly flexible ensemble models were prone to overfitting in our relatively small single-center dataset. Although such algorithms can capture complex nonlinear relationships and high-order interactions, this flexibility may also allow them to fit sample-specific noise and idiosyncratic patterns that are not reproducible in unseen patients. In contrast, the SVM demonstrated a substantially smaller decline in discrimination, from an AUC of 0.912 in the training cohort to 0.849 in the validation cohort. In the context of the six predictors retained by LASSO, the regularized SVM configuration used in our study may have provided a more favorable bias–variance balance and a smoother decision boundary, thereby limiting excessive adaptation to the training data. These findings emphasize that near-perfect training performance should not be interpreted as evidence of superior generalizability, particularly when complex “black-box” models are applied to modest-sized clinical datasets. Accordingly, our selection of SVM as the final model was driven primarily by its superior validation performance and comparatively limited training–validation degradation rather than by training-set discrimination alone. The subsequent SHAP analysis further improved model transparency by illustrating the contribution and direction of individual predictors to model-estimated LNM risk.

Despite these findings, several limitations should be acknowledged. First, this was a single-center retrospective study with a modest sample size of 232 patients, including 163 patients in the training cohort and 69 patients in the internal validation cohort. The outcome distribution was moderately imbalanced (62.5% LNM vs. 37.5% non-LNM), which may have contributed to the relatively high sensitivity but lower specificity observed in several models and may limit the stability of performance estimates. Second, the extent of thyroidectomy and cervical lymph-node dissection was not completely uniform. Because LNM status was determined from surgically resected lymph nodes, differences in nodal sampling may have resulted in under-detection of occult metastases and potential outcome misclassification. Third, the requirement for available *BRAF*^V600E^ testing may have introduced selection bias. Although LNM prevalence did not differ significantly between the final analytic cohort and patients without available *BRAF* testing (62.5% vs. 60.3%, P = 0.567; **Supplementary Table 2**), residual differences in other measured or unmeasured clinical characteristics cannot be excluded. Fourth, standardized preoperative ultrasound features and Hashimoto’s thyroiditis were not available for consistent retrospective ascertainment and therefore were not included as candidate predictors. Finally, several predictors were derived from postoperative pathological assessment, limiting direct application in a purely preoperative setting. Larger prospective multicenter studies incorporating standardized imaging, standardized surgical and nodal assessment, and additional clinically relevant variables are warranted to confirm the robustness and generalizability of the model.

In summary, we developed and internally validated an SVM-based model for LNM risk stratification in PTC. The model demonstrated favorable internal discrimination and may provide a basis for individualized postoperative risk assessment; however, its clinical generalizability requires confirmation in larger, prospectively collected multicenter cohorts.

## Conclusion

Our findings demonstrate that the SVM-based model achieved favorable discrimination for LNM prediction in PTC patients, with a validation AUC of 0.849 (95% CI, 0.756–0.934). By integrating six key clinical and molecular markers, this model provides an objective framework for individualized LNM risk stratification and may support personalized clinical management. Further prospective multicenter validation is warranted before routine clinical implementation.

## Data Availability

The clinical datasets supporting the findings of this study are available from the corresponding author upon reasonable request and with permission from the participating institution, subject to ethical approval and applicable data-use agreements.
