# Supplementary material for: Machine learning-based prediction of lymph node metastasis in papillary thyroid carcinoma: an interpretable multidimensional model
Source: Front Endocrinol (Lausanne). 2026 Sep 28;17:1866805. doi: 10.3389/fendo.2026.1866805 (PMC13654693; doi:10.3389/fendo.2026.1866805)
Supplement: Supplementary file 1 [file Table1.docx]

**Supplementary Table S1. Hyperparameter search spaces and selected values for machine-learning models.**

| **Model** | **Hyperparameter** | **Candidate values explored** | **Selected value** |
| --- | --- | --- | --- |
| **LR** | — | No grid search; baseline model | — |
| **DT** | max_depth | 3, 5, 10, None | None |
|  | min_samples_split | 5, 10, 20 | 5 |
|  | max_features | 'sqrt', None | 'sqrt' |
|  | ccp_alpha | 0.0, 0.01, 0.1 | 0.01 |
| **RF** | n_estimators | 50, 100, 150, 200, 250, 300, 350, 400, 450 | 50 |
|  | max_features | 2* | 2 |
| **XGBoost** | learning_rate | 0.01, 0.1, 0.2 | 0.2 |
|  | max_depth | 3, 5, 10 | 3 |
|  | n_estimators | 50, 100, 200 | 50 |
|  | subsample | 0.6, 0.8, 1.0 | 0.6 |
| **LightGBM** | learning_rate | 0.1, 0.2 | 0.1 |
|  | num_leaves | 31, 50, 100 | 31 |
|  | n_estimators | 100 | 100 |
|  | subsample | 0.6, 0.8, 1.0 | 0.6 |
|  | colsample_bytree | 0.6, 0.8, 1.0 | 1.0 |
| **SVM** | C | 0.1 | 0.1 |
|  | kernel | 'linear', 'rbf' | 'rbf' |
|  | gamma | 'scale', 'auto', 0.01 | 0.01 |
|  | degree | 2, 3, 4 | 2 |
| **ANN** | hidden_layer_sizes | (25,), (50,), (100,), (10,10), (50,50), (100,100), (50,50,50) | (10,10) |
|  | activation | 'relu', 'tanh', 'logistic' | 'tanh' |
|  | alpha | Fixed during model fitting† | 0.001 |
|  | batch_size | Fixed during model fitting† | 16 |
|  | learning_rate_init | Fixed during model fitting† | 0.005 |

**Table note:** Grid search was conducted using 5-fold cross-validation in the training cohort, with AUC as the optimization metric. LR was used as the baseline model without grid-search optimization. For RF, max_features was defined according to the number of selected predictors; with six retained predictors, the evaluated value was 2.
