# Supplementary material for: Machine learning-based prediction of lymph node metastasis in papillary thyroid carcinoma: an interpretable multidimensional model
Source: Front Endocrinol (Lausanne). 2026 Sep 28;17:1866805. doi: 10.3389/fendo.2026.1866805 (PMC13654693; doi:10.3389/fendo.2026.1866805)
Supplement: Supplementary file 2 [file Table2.docx]

**Supplementary Table S2.** Comparison of pathological lymph node metastasis between the final analytic cohort and patients excluded because of unavailable *BRAF*^V600E^ testing

| **Group** | **N** | **LNM,**  **n (%)** | **Non-LNM,**  **n (%)** | ***P*** |
| --- | --- | --- | --- | --- |
| Final analytic cohort (*BRAF* testing available) | 232 | 145 (62.5%) | 87 (37.5%) | 0.567 |
| Excluded because *BRAF* testing was unavailable | 468 | 282 (60.3%) | 186 (39.7%) |  |

**Note:** Among the 729 patients remaining after exclusion of non-malignant tumors and cases with unclear pathological diagnoses, 468 were excluded because *BRAF*^V600E^ testing was unavailable, leaving 261 patients with available *BRAF* testing. A further 29 patients were excluded because of incomplete clinical or biochemical data, resulting in the final analytic cohort of 232 patients. The present comparison therefore evaluates pathological LNM prevalence between the final analytic cohort and patients excluded specifically because of unavailable *BRAF* testing. LNM, lymph node metastasis. The between-group difference was assessed using Pearson’s χ² test. A two-sided P < 0.05 was considered statistically significant.
