# Supplementary material for: Machine learning-based prediction of lymph node metastasis in papillary thyroid carcinoma: an interpretable multidimensional model
Source: Front Endocrinol (Lausanne). 2026 Sep 28;17:1866805. doi: 10.3389/fendo.2026.1866805 (PMC13654693; doi:10.3389/fendo.2026.1866805)
Supplement: Supplementary file 3 [file Table3.docx]

**Supplementary Table S3.** Ablation analysis evaluating the incremental contribution of *BRAF*^V600E^ mutation status to the SVM model.

| **Model** | **Predictors** | **Validation**  **AUC**  **(95% CI)** | **Accuracy** | **Sensitivity** | **Specificity** | **ΔAUC (Full − Reduced)** | ***P*** |
| --- | --- | --- | --- | --- | --- | --- | --- |
| **Full SVM model** | *BRAF*^V600E^, tumor size, capsular invasion, ETE, multifocality, TSH | 0.849 (0.756–0.934) | 0.768 | 0.860 | 0.615 | 0.007 | 0.671 |
| **Reduced SVM model without *BRAF*** | Tumor size, capsular invasion, ETE, multifocality, TSH | 0.843 (0.747–0.928) | 0.797 | 0.860 | 0.692 | Reference | — |

**Note:** AUC, area under the receiver operating characteristic curve; CI, confidence interval; ETE, extrathyroidal extension; SVM, support vector machine; TSH, thyroid-stimulating hormone. Both models used the same training/validation partition and the same 5-fold grid-search framework. After removal of *BRAF*^V600E^, the reduced SVM was re-tuned exclusively in the training cohort. Individual AUC 95% CIs were estimated using 1,000 bootstrap resamples. The paired difference in validation AUC was assessed using DeLong’s test. The optimal hyperparameters for the reduced SVM were C = 0.1, degree = 2, gamma = ‘auto’, and kernel = ‘rbf’. Classification metrics were calculated at a probability threshold of 0.50.
